# Hyperglycemia induces endoplasmic reticulum stress-dependent CHOP expression in osteoblasts

**DOI:** 10.3892/etm.2013.978

**Published:** 2013-02-27

**Authors:** WEI LIU, XIAOQING ZHU, QIAN WANG, LINLIN WANG

**Affiliations:** 1 Department of Prosthetics, Stomatology Hospital, School of Medicine, Zhejiang University, Hangzhou 310058, P.R. China; 2 Department of Basic Medical, School of Medicine, Zhejiang University, Hangzhou 310058, P.R. China

**Keywords:** diabetic osteoporosis, C/EBP-homologous protein, endoplasmic reticulum stress, osteoblasts

## Abstract

Diabetic osteoporosis is a metabolic bone disease responsible for global health problems. Hyperglycemia induces osteopenia, increases bone fragility and unbalances the coupling of osteoblasts and osteoclasts. The mechanism is, however, unknown. For the purpose of this study, we hypothesized that hyperglycemia destroys endoplasmic reticulum (ER) homeostasis, activates C/EBP-homologous protein (CHOP) and induces osteoblast apoptosis under diabetic conditions. Diabetic rats were created by injecting streptozotocin (STZ) 65 mg/kg intraperitoneally and their osteoblasts were cultured under high-glucose medium *in vitro*. The bone mineral density (BMD) and pathological changes of the rats’ femurs were observed. The expression of CHOP in osteoblasts was assayed using immunohistochemistry and western blot analysis. Six weeks after diabetic model establishment, a significant decrease was found in the BMD of the diabetic rat femurs, and the numbers of osteoblasts under cortical bone were also reduced. The expression of the ER stress regulator CHOP in osteoblasts of diabetic rats or high-glucose medium was also elevated (P<0.01). The present results demonstrated that hyperglycemia elevated the expression of CHOP and finally led to osteoporosis. This suggested that elevating the expression of CHOP may play a role in diabetic osteoporosis.

## Introduction

Patients with diabetes appear to have a higher risk of hip and upper extremity fracture compared with non-diabetics (1). Diabetes mellitus (DM) influences bone metabolism and contributes to bone loss (2). A histomorphometric study in DM has described low recruitment of osteoblasts and diminished mineral apposition rates with no mineralization defects (3). Animal studies have also demonstrated that bone formation was impaired in a diabetic mouse model during tibial distraction osteogenesis (4). In a bone-loss mouse model, DM also decreased osteoclastogenesis, reduced bone formation and enhanced osteoblast apoptosis (5). The mechanism of DM-related osteoporosis has yet to be elucidated.

Under hyperglycemia, the unbalanced coupling of bone resorption and formation in remodeling promotes excess bone resorption (6). Thus, DM causes a more persistent response, greater loss of attachment and more alveolar bone resorption and impaired new bone formation. The key mechanism of osteoblast-osteoclast coupling imbalance is the regulation of osteoblast cellular apoptosis (7). Under normal conditions, stress within the ER triggers an adaptive cellular mechanism known as the unfolded protein response (UPR) that attempts to return the cell to homeostasis under hyperglycemia. If hyperglycemia persists and cellular homeostasis is not restored, the ER stress response initiates cell death stimuli that lead to ER stress-induced apoptosis (8). Initiation of ER stress-induced apoptosis through ER stress response signaling involves transcriptional activation of the C/EBP-homologous protein (CHOP) (9,10). CHOP is induced in response to cellular stress, especially ER stress, and is involved in the ER stress-induced apoptosis pathway (11). In this study, it was hypothesized that hyperglycemia may induce CHOP-mediated ER stress apoptosis in osteoblasts and result in unbalanced coupling of osteoblasts and osteoclasts and ultimately lead to diabetic osteoporosis.

The aim of the present study was to explore the pathological changes and the CHOP activity in diabetic rats femurs and high-glucose cultured osteoblasts.

## Materials and methods

### Animal model induction and bone mineral density (BMD) observation

Twenty male Sprague Dawley (SD) rats (purchased from the Experiment Animal Center of Zhejiang University) were divided into two groups (n=10) at random. The rats in the diabetic group were fasted for 10 h and injected intraperitoneally with streptozotocin (STZ) (Alexis Corporation, Switzerland) 65 mg/kg to induce diabetes. The other 10 rats formed the control group and they were fasted for 10 h and injected with 0.9% saline. The rats with blood glucose >16 mmol/l 48 h after injection were considered diabetic. All procedures were approved by the Zhejiang University Institutional Animal Care and Use Committee.

Six weeks after diabetes was induced, all rats were anesthetized, their thoracic cavities opened and perfused intracardially with normal saline. Following saline perfusion, the animals were perfused with 300–400 ml fixative containing 4% paraformaldehyde in 0.1 mol/l phosphate buffer (pH 7.4). After perfusion, both femurs were extracted. The BMD of the left femurs was determined using a small animal radiophotography system (Faxitron model MX20; Tucson, AZ, USA). The right femurs were used for hematoxylin and eosin (H&E) staining and immunohistochemistry assay.

### Osteoblast culture and western blot analysis

Primary osteoblasts were derived from newborn rat calvaria as previously described (24). Cells were seeded in 6-, 24- or 96-well plates in Dulbecco’s modified Eagle’s medium (DMEM; Invitrogen, Carlsbad, CA, USA) supplemented with antibiotics (penicillin 100 U/ml and streptomycin 100 *μ*g/ml) and 15% fetal bovine serum (FBS) and incubated at 37°C in a humidified atmosphere of 5% CO_2_ and 95% air. Cells were used between the second and the fourth passages. The third passage cells were divided into a normal medium group and a high-glucose medium group (concentrations of glucose 100 mmol/l). The osteoblasts of both groups were cultured for 48 h and washed twice with phosphate-buffered saline (PBS). Ten wells of osteoblasts for each group were used for CHOP western blot analysis.

The cells were prepared in lysis buffer and centrifuged (12,000 × g for 10 min at 4°C) to remove cellular debris. The protein concentration was determined by the Lowry method using a Bio-Rad DC protein assay kit (Bio-Rad, Hercules, CA, USA). The lysates containing equal amounts of protein (50 *μ*g) were resolved using 8–10% SDS-polyacrylamide gel electrophoresis and transferred onto Millipore (Billerica, MA, USA) nitrocellulose membranes. The reactions were stopped with a solution containing 5% skimmed milk in Tris-buffered saline with 0.05% Tween-20 (TBST) for 1 h at room temperature and treated with CHOP antibodies (1:2,000, Santa Cruz Biotechnology Inc., Santa Cruz, CA, USA) in TBST overnight at 4°C, washed for 1 h with TBST and further probed with secondary horseradish peroxidase (HRP; 1:2,000) in TBST for 1 h at room temperature. The immune complexes were visualized using an enhanced chemiluminescence (ECL) detection system according to the manufacturer’s protocols. The densitometric analysis of the bands was assayed using Quantity One (Bio-Rad).

### H&E staining and immunohistochemistry assay

The remaining right femurs were fixed in the same fixative for 4 h and placed in 30% phosphate-buffered sucrose until the tissue sank. Sections 12 *μ*m thick were cut on a freezing microtome through the coronary planes of the proximal femur for H&E staining and diaminobenzidine (DAB) immunohistochemical staining.

Femur sections were rinsed in 0.01 M PBS and mounted onto 0.02% poly-l-lysine-coated slides. The ABC system was used with DAB as the chromagen. Tissue sections were first washed in PBS and incubated in 1% bovine serum albumin (BSA) for 30 min. Tissues were incubated overnight at 4°C in the medium of PBS with CHOP antibody (1:100) plus 1% BSA. Control sections were incubated in PBS and 1% BSA. The following day, the sections were incubated in a biotinylated goat-anti-mouse secondary antibody (diluted 1:200 in PBS, Boster Biotechnology Company, Wuhan, China) and subsequently in an avidin-HRP solution. Immunolabeling was visualized with 0.05% DAB plus 0.3% H_2_O_2_ in PBS. The sections were dehydrated through ethanol and xylene before mounting.

Immunohistochemistry results were analyzed using CHOP-positive osteoblast in femur per mm^2^ of two groups of rats under a Nikon microscope (Nikon E600, Nikon Company, Japan) at final magnifications of ×400.

### Statistical analysis

CHOP-positive cells in each visual field under the microscope were counted at ×200 magnification. The data represent the means ± SD. The differences were evaluated by analysis of two-tailed t-tests. P<0.05 was considered to indicate a statistically significant result. All computations were performed using SPSS 15.0 (SPSS Inc., Chicago, IL, USA).

## Results

### Body weight, blood glucose and proximal femoral BMD analysis

**Figure 1:**
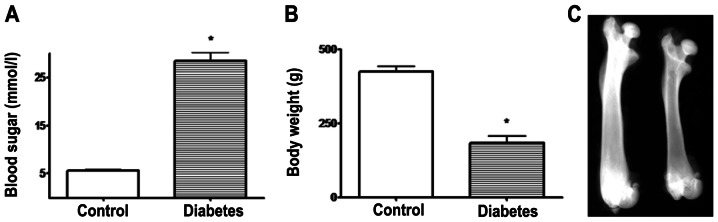
Body weight, blood glucose and proximal femoral bone mineral density (BMD) analysis in the two groups six weeks after streptozotocin (STZ) injection. (A) Blood glucose in the two groups; (B) Body weight in the two groups.^*^P<0.01, vs. control. (C) X-ray images of a left femur isolated from the two groups. Left, control rat; Right, diabetic rat showed more bone loss and reduced length.

Initially, the body weight and blood glucose showed no significant differences (P>0.05) in both groups of rats before STZ injection. After 6 weeks, the diabetic rats had a significantly higher blood glucose and lower body weight when compared with the control group rats (P<0.01). Compared with non-diabetic control rats, diabetic rats showed a decrease in femoral BMD (−15.4±2.3% vs. control, P<0.05, Fig. 1).

### H&E staining and immunohistochemistry assay

**Figure 2:**
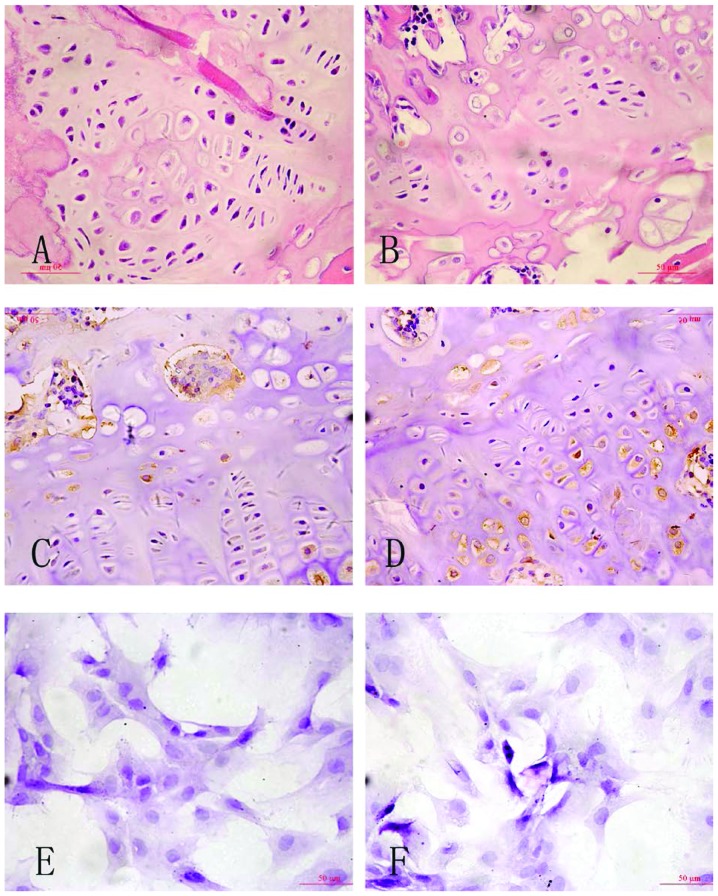
Hematoxylin and eosin (H&E) staining and immunohistochemistry assay. H&E staining of the proximal femur tissues; magnification, ×400 (A–B). (A) A control rat. (B) A diabetic rat showed more osteoclasts that clumped together and reduced cortical bone. The overall growth plate architecture was dominated by hypertrophic chondrocytes, rare new bone formation with fewer proliferative and chondroblastic cells. Light photomicrographs of C/EBP-homologous protein (CHOP) immunohistochemical staining of the osteoblasts from proximal femur (C–D) and cultured osteoblasts (E–F) in the two groups. Cells that stained positive for CHOP showed buff-coloured granules with DAB staining. (C) A control rat showed few CHOP-positive osteoblasts. (D) A diabetic rat showed more CHOP-positive osteoblasts in the overall growth plate. (E) Control conditions (normal medium) showed few CHOP-positive osteoblasts. (F) Hyperglycemia condition showed more CHOP-positive osteoblasts.

Osteoblasts were identified by a central lucency, eccentric nucleus and basophilic cytoplasmic stain. Osteoclasts were identified as single or multi-nucleated cells demonstrating foamy cytoplasm. Six weeks after inducing diabetes, the proximal femur H&E staining demonstrated a greater number of osteoclasts that clumped together, reduced cortical bone and a deteriorated bone micro-architecture in diabetic rats. The overall growth plate architecture was dominated by hypertrophic chondrocytes, with fewer proliferative and chondroblastic cells as compared with control rats. The femur of control rats showed a normal shape with intact cartilage, qualitatively equivalent cortical bone and a well-organized bone matrix composed of trabecular bone compared with the diabetic rats (Fig. 2).

CHOP immunoreactivity was visualized in a granular immunostain pattern in the nucleus. Caspase-12 immunohistochemistry staining positive cells with DAB staining, showed buff-colored granules in the cytoplasm. Quantitative analysis for the number and optical density of CHOP-positive cells with DAB immunostaining showed significant increases in osteoblast cells in diabetic rats and under high-glucose medium *in vitro* (P<0.05, Fig. 2 and Table I).

The number of CHOP-positive cells and the optical density was significantly increased in the rats of the diabetic group (P<0.05). Similarly, the number of positive cells and the optical density was upregulated in the osteoblasts of high-glucose medium compared with that of the normal medium group (P<0.05).

### Western blot analysis

**Figure 3:**
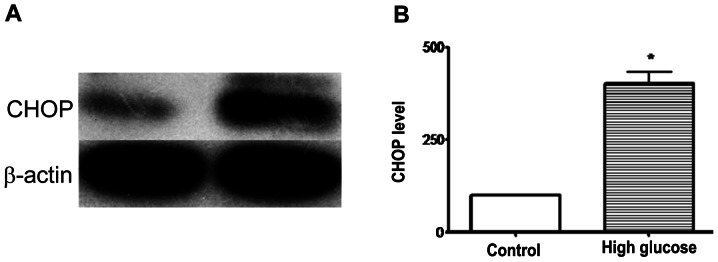
Western blot analysis for C/EBP-homologous protein (CHOP) in osteoblasts of both groups. (A) Expression levels of the CHOP protein in normal medium and hyperglycemia medium. Osteoblasts were assessed by western blot analysis and anti-beta-actin antibody (as a loading control). (B) The data are presented as the mean ± SD; ^*^P<0.01, vs. control rats.

CHOP proteins in osteoblast cells were detected as single bands migrating ∼27 kDa. The densitometric analysis of the bands for CHOP revealed a significant increase in relative protein content (451.4±32.6%) in high-glucose medium osteoblasts when compared with those from the normal medium group (100.00%; P<0.05, Fig. 3). This suggested that CHOP was activated in osteoblasts under high-glucose medium.

## Discussion

DM is a group of pandemic debilitating metabolic diseases featuring chronic hyperglycemia which results from defective insulin secretion and/or insulin actions. Such chronic hyperglycemia typically elicits dysfunction and failure of various organs, particularly the eyes, kidneys, heart and nerves (12–15). In addition, DM has been found to be associated with metabolic bone diseases, osteoporosis and low-impact fractures (16). Patients with diabetes are at greater risk of fractures due to their low BMD. Lower BMD is explained by insulinopenia and hyperglycemia, which impair bone formation (17). Despite the discrepancies between BMD and fracture rates, clinical trials uniformly support the fact that new bone formation and bone microarchitecture and bone quality are altered in both types of diabetes (18,19). In the present study, STZ-treated rats showed a decrease in femoral BMD compared with normal control rats. The femur of diabetic rats showed reduced cortical bone, deteriorated bone micro-architecture, a decrease in the trabecular width in the epiphysis and metaphysis and increased apoptosis of osteoblasts when compared with the control rats.

ER is the organelle where secretory and membrane proteins are synthesized and folded. In certain severe situations of ER stress, however, the protective mechanisms activated by the UPR are not sufficient to restore normal ER function and cells die by apoptosis (20). Hyperglycemia accumulates the misfolded proteins and induces alterations in Ca^2+^ homeostasis (21). The UPR in osteoblasts alleviate ER stress by upregulation of chaperones (such as BiP/GRP78) and degradation of misfolded proteins (22). However, under persistent hyperglycemia conditions, the UPR triggers apoptotic cell death (23). Microarray studies revealed that CHOP is one of the most highly inducible genes during ER stress and increases during ER stress during apoptosis (24). Overexpression of CHOP and microinjection of CHOP protein have been reported to play a key role in apoptosis by regulating the expression of proapoptotic proteins (25). Besides the possible mechanism that overexpression of CHOP in the bone microenvironment may impair the function of osteoblasts leading to osteopenia (26), CHOP may act as a dominant-negative inhibitor of C/EBP and prevent osteoblast differentiation (27). In the current diabetic osteoporosis rat model, diabetes significantly elevated the expression of CHOP in osteoblast cells. CHOP promotes osteoblast reversal of translational repression caused by the UPR to facilitate progress of apoptosis *in vivo* and *in vitro*. It may be hypothesized, therefore, that high blood sugar status, contributing to the high expression of CHOP, facilitates the progress of apoptosis, which upsets the osteoblast-osteoclast balance, leading to bone disorders and the development of diabetic osteoporosis. The relationship between CHOP expression and osteoclasts remains to be fully elucidated.

## Figures and Tables

**Table I:** Comparison of C/EBP-homologous protein (CHOP)-positive cells and optical density in both groups (mean ± SD).

Group	No. of positive cells	Optical density
Control rats	8.3±2.1/mm^2^	102.4±12.1
Diabetic rats	25.4±7.6/mm^2^^a^	180.3±17.4^a^
Osteoblasts in normal medium	3.9±1.2/mm^2^	112.1±13.8
Osteoblasts in high-glucose medium	43.7±11.4/mm^2^^b^	176.1±15.3^b^

a P<0.05, vs. control rats,

b P<0.05, vs. osteoblasts in normal medium.
